# Shock-transformation of whitlockite to merrillite and the implications for meteoritic phosphate

**DOI:** 10.1038/ncomms14667

**Published:** 2017-03-06

**Authors:** C. T. Adcock, O. Tschauner, E. M. Hausrath, A. Udry, S. N. Luo, Y. Cai, M. Ren, A. Lanzirotti, M. Newville, M. Kunz, C. Lin

**Affiliations:** 1 Department of Geoscience, University of Nevada, Las Vegas, 4505 South Maryland Parkway, Las Vegas, Nevada 89154, USA; 2 High Pressure Science and Engineering Center, University of Nevada, Las Vegas, 4505 South Maryland Parkway, Las Vegas, Nevada 89154, USA; 3 LSPM-CNRS, Institut Galilée, Université Paris 13, Nord, 99, av. J. B. Clément, 93430 Villetaneuse, France; 4 Key Laboratory of Advanced Technologies of Materials, Ministry of Education, Southwest Jiaotong University, Chengdu, Sichuan 610031, China; 5 The Peac Institute of Multiscale Sciences, Chengdu, Sichuan 610031, China; 6 CAS Key Laboratory of Materials Behavior and Design, Department of Modern Mechanics, University of Science and Technology of China, Hefei, Anhui 230027, China; 7 GeoScienceEnviro Center for Advanced Radiation Sources, University of Chicago, Advanced Photon Source, Argonne National Laboratory, Argonne, Illinois 60439, USA; 8 Lawrence Berkeley National Laboratory, Advanced Light Source, University of California, Berkeley, Berkeley, California 94720, USA; 9 High Pressure Collaborative Access Team (HPCAT), Geophysical Laboratory, Carnegie Institution of Washington, Argonne, Illinois 60439, USA

## Abstract

Meteorites represent the only samples available for study on Earth of a number of planetary bodies. The minerals within meteorites therefore hold the key to addressing numerous questions about our solar system. Of particular interest is the Ca-phosphate mineral merrillite, the anhydrous end-member of the merrillite–whitlockite solid solution series. For example, the anhydrous nature of merrillite in Martian meteorites has been interpreted as evidence of water-limited late-stage Martian melts. However, recent research on apatite in the same meteorites suggests higher water content in melts. One complication of using meteorites rather than direct samples is the shock compression all meteorites have experienced, which can alter meteorite mineralogy. Here we show whitlockite transformation into merrillite by shock-compression levels relevant to meteorites, including Martian meteorites. The results open the possibility that at least part of meteoritic merrillite may have originally been H^+^-bearing whitlockite with implications for interpreting meteorites and the need for future sample return.

## Abstract (web summary)

Quantifying the amount of water in meteorites remains challenging, with minerals the key to understanding water contents. Here, Adcock *et al*. perform shock experiments on H^+^-bearing whitlockite demonstrating that it may transform into anhydrous merrillite, which is commonly found in Martian meteorites.

Meteorites preserve evidence of processes ranging from the formation of the solar system to the origin of life on Earth and the potential for extraterrestrial habitability^1^,^2^,^3^,^4^. For numerous solar system bodies, meteorites represent the only physical samples available for study on Earth. This includes Mars, where paragenetic (petrographic) relationships of minerals, total water or volatile content, and rare Earth elements (REEs) distribution in minerals of Martian meteorites have all shed light on Martian planetary processes such as mantle and crustal evolution, planetary volatile contents and even the potential for past Martian life^1^,^5^,^6^,^7^,^8^,^9^,^10^,^11^,^12^,^13^.

One mineral of particular interest in meteorites is the phosphate mineral merrillite (Ca_9_Na[Fe,Mg][PO_4_]_7_). Merrillite is the anhydrous end-member of the merrillite–whitlockite solid solution series^14^, with whitlockite (Ca_9_[Fe,Mg][HPO_4_][PO_4_]_6_) being the hydrogenated end member. Merrillite can be produced by dehydrogenation (that is, devolatilization) of whitlockite in the laboratory through heating to 1,000–1,100 °C (refs 14, 15, 16), although at least partial transformation may be possible at temperatures as low as 700 °C (ref. 15). The mineral occurs in most meteorite types including chondrites, achondrites, pallasites and both Martian and lunar meteorites where it is a major carrier of REEs^17^,^18^,^19^,^20^,^21^,^22^. Natural merrillite is generally igneous, although it can occur as a metamorphic product in chondritic meteorites^17^,^18^,^23^. Along with chlorapatite (Ca_5_[PO_4_]_3_[Cl]), merrillite is one of the two most commonly occurring phosphates in meteorites^17^,^20^,^23^,^24^. In fact, the mineral is typically the dominant phosphate phase in shergottite Martian meteorites, the most common type of Martian meteorites, where it is two to three times more abundant than chlorapatite, suggesting merrillite may be the dominant igneous phosphate on Mars as well^1^,^20^,^25^,^26^. The predominance of merrillite in Martian materials also makes the mineral a potential source of bioessential phosphate on Mars and thus merrillite is of astrobiological importance^1^. This is all in contrast to Earth, where merrillite does not generally occur (except as a minor solid solution component within whitlockite^14^), fluorapatite is the dominant primary phosphate mineral and REEs are hosted predominantly by terrestrial igneous silicates^27^,^28^,^29^. Whitlockite, the nearest terrestrially occurring analogue of merrillite, has not been confirmed in any type of meteorite (despite some historical nomenclature confusion in the literature)^16^,^17^,^30^ and on Earth it is a rare mineral typically associated with pegmatites, or is biogenic^14^,^31^,^32^,^33^. These contrasting roles of merrillite in Martian and other meteorites compared with Earth suggests that the mineral holds important clues to processes that do not generally occur in terrestrial geologic settings.

A significant property of igneous phosphate minerals is that they often form in the late stages of magma evolution, including just before magma degassing to the surface^7^,^17^,^34^,^35^. For this reason, the volatile content and intergrowth relationships of phosphate minerals in Martian meteorites have been used as indicators of late-stage melt evolution. Thus, the pervasive occurrence and anhydrous nature of merrillite have previously been interpreted as evidence of relatively dry late-stage Martian melts^5^,^6^,^7^,^13^,^14^,^30^,^36^.

However, recent observations of OH contents of chlorapatite in shergottite Martian meteorites suggest relatively high water content in melts during phosphate crystallization. This is despite the coexistence and even intergrowth of merrillite and apatite in the same meteorites^11^,^26^,^30^. These observations have raised the question as to why merrillite rather than whitlockite forms in a melt with available H_2_O at the time of phosphate crystallization (Supplementary Note 1)^14^,^30^. One possibility, and the hypothesis that we are testing in this work, is that shock has devolatilized what was, in part or whole, whitlockite into merrillite.

All meteorites have experienced some degree of shock. Peak shock pressures for chondritic and achondritic meteorites can range from 3 to >80 GPa^37^,^38^. Martian meteorites, being the products of ejecta launched from Mars by impacts, are constrained to a narrower peak shock range of 5 to ∼55 GPa^39^,^40^. The lower boundary is the minimum impact energy required to accelerate ejecta beyond Martian escape velocity^39^,^40^. The upper limit is the product of multiple factors including ejecta size, large impact event statistics and Mars/Earth transit times^39^,^40^,^41^.

Shock compression induces deformation, phase transformations and chemical reactions in meteorites. For instance, tuite (γ-Ca_3_[PO_4_]_2_) is a shock-generated phosphate mineral, which has been identified in both chondritic and achontritic meteorites^42^,^43^. Devolatilization of amphiboles in a Martian meteorite has also been documented^44^ and maskelynite (shock-induced amorphized feldspar) occurs in many meteorites including in 93% of basaltic Martian meteorites^45^. Therefore, whenever making interpretations from meteorite mineralogy, the effects of shock must be considered.

In addition to pressure, heating also results from a shock event. Bulk rock heating from shock can be modest, for example, Martian merrillite-bearing meteorites experience <400 °C (ref. 42). However, localized heating during a shock event can lead to temperatures as high as the rock liquidus^42^,^46^. These localized ‘hotspots' occur at locations of low-shock impedance where a larger fraction of shock energy is converted to heat rather than compression. Sources of hotspot formation include frictional heating between minerals of different impedances, collapse of voids or fluid inclusions or breakdown of hydrous minerals upon shock^42^,^46^. Whitlockite/merrillite has a lower shock impedance than the major rock-forming minerals in meteorites^20^. Hence, shock-induced heating where merrillite occurs is anticipated to be higher than bulk rock heating, with ‘hotspots' that can well exceed the 1,000 °C required to transform whitlockite into merrillite. This, combined with possible pressure-effects, suggests that shock could potentially devolatilize whitlockite to merrillite.

Despite the importance of merrillite, and phosphate minerals in general, to the best of our knowledge, no laboratory shock experiments on these or any extraterrestrially relevant phosphate minerals have been performed. Therefore, to test the hypothesis that meteoritic merrillite with similar compositions to those observed in Martian shergottite meteorites co-existing with high OH^−^ chlorapatite may be shock transformed from whitlockite, we conduct a set of shock experiments on synthetic Mg-whitlockite in the lower range of peak pressures (∼7 to 20 GPa) experienced by Martian meteorites^40^. Recovered samples are analysed by synchrotron X-ray diffraction (XRD). Our results show that merrillite is produced from whitlockite during experimental shock events. These findings have potential implications for meteorite studies, especially where minerals, such as merrillite, are used to interpreted parent body volatile abundances.

## Results

### Overview

As no single laboratory experiment can encompass the full complexity of a natural impact event, we performed a set of four shock experiments (GG092 through GG095) focused on three specific shock-induced processes, which could potentially transform whitlockite to merrillite. These processes were heating through void collapse (experiments GG092 and GG094)^47^, frictional heating due to mineral impedance contrasts (experiment GG093) and shock compression effects (experiments GG094 and GG095)^48^. The general experimental conditions are listed in Supplementary Table 1 and analytical results are detailed below.

### Void collapse

Experiment GG092 was performed using whitlockite powder (0.45–10 μm size fraction) with a void of ∼20 μm intentionally put in the sample. For comparison, GG094 was an identical experiment run without a void in the sample beyond the powder packing interstices, which were on the order of a few micrometres. Peak shock pressures, duration and velocities were calculated from measured flyer plate velocities during experiments, the impedances of flyers, drivers and their thicknesses using impedance match equations^36^ (Table 1 and Supplementary Table 1). Peak shock pressures for both experiments were calculated at ∼20 GPa (Table 1). Analysis of the recovered samples by synchrotron XRD (see Methods) indicates the presence of merrillite in both samples (Table 1 and Supplementary Figs 1 and 2). Experiment GG092, which contained the void space, yielded a nearly 90% transformation of whitlockite to merrillite and an additional, previously unknown, Ca/Mg-phosphate phase structurally related to hurlbutite (CaBe_2_[PO_4_]_2_) (Table 1, Supplementary Fig. 1 and Supplementary Data 1). Experiment GG094, without the void space, achieved about 12% whitlockite to merrillite transformation and contained no additional phase (Supplementary Fig. 2 and Supplementary Data 1).

### Impedance contrast heating

**Figure 1:**
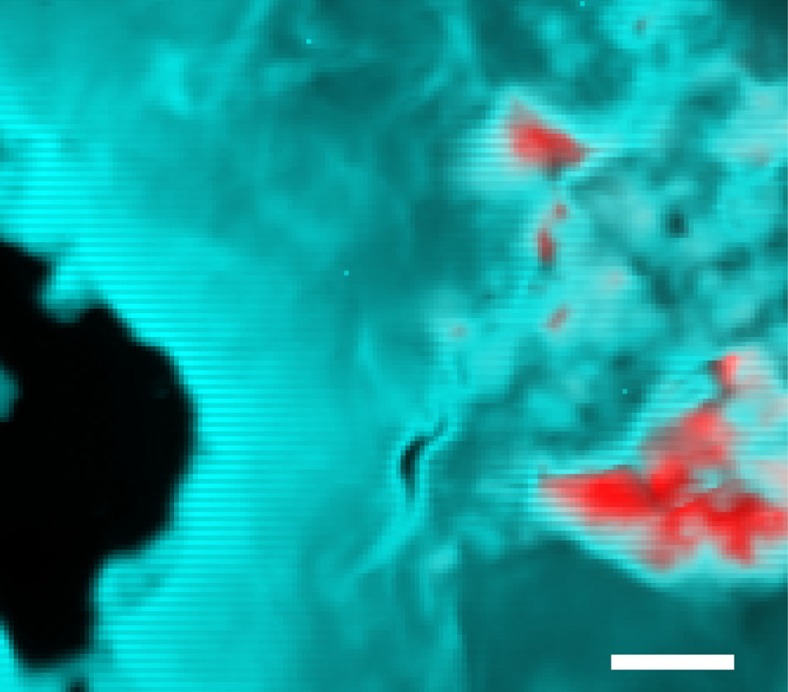
X-ray fluorescence map of recovered sample GG093 showing a whitlockite single crystal grain in copper matrix. Red=Ca (whitlockite/merrillite), Blue=copper. Diffraction images were taken in a grid scan over the exposed phosphate grains. At the contact between matrix and the crystal ∼35% of whitlockite was transformed into merrillite (see Fig. 2). Further inward the amount of merrillite is smaller and the innermost kernel of the former crystal is highly strained polycrystalline whitlockite; 1 pixel=2 × 2 μm^2^. Map taken at beamline 13-IDE at the APS, Argonne National Laboratory. Colour-modified image. Raw image data appear as Supplementary Fig. 3. Scale bar, 50 μm.

**Figure 2:**
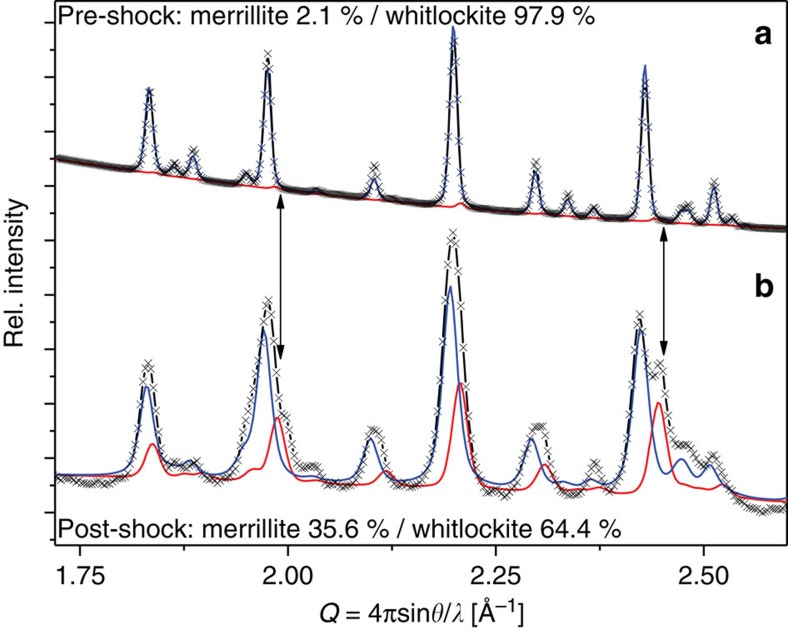
Pre- and post-shocked synthetic whitlockite. Black crosses, collected diffraction data. Blue line, modelled pattern of whitlockite. Red line, modelled pattern of merrillite. We chose *Q* over conventional 2*θ*, because the two patterns were collected at different X-ray wavelengths (see Methods). (**a**) Diffraction pattern for pre-shocked synthetic Mg-whitlockite (UNLV Batch B4). Sample is whitlockite with approximately 2% merrillite, consistent with previous work^14^,^16^. Sample grain size was approximately 300 nm. (**b**) Diffraction pattern for the same synthetic Mg-whitlockite material recovered from shock experiment GG093. The shocked sample contains ∼35 mass % merrillite. Overall diffraction peaks of the shock-recovered sample are broader than in the starting material because average grain size was reduced to 45–60 nm (based on diffraction peak width analysis).

Shock experiment GG093 was conducted on synthetic Mg-whitlockite crystals (∼50–75 μm diameter) embedded in a powdered Cu matrix, to examine the effects of frictional heating from impedance contrast. Copper was used because it has a similar bulk impedance as shergottitic basalt (*C*=3.9 versus 4.3 km s^−1^ and *S*=1.5 versus 1.6) in the examined pressure regime^49^,^50^,^51^,^52^. Frictional motion of whitlockite relative to copper was approximately 100 μm during the experiment (roughly comparable to the whitlockite crystal diameter). The peak shock pressure of the experiment was calculated at 19±2 GPa. The recovered sample was analysed by synchrotron micro-XRD and X-ray fluorescence mapping at beamline 13-IDE, of the Advanced Photon Source (APS) at Argonne National Laboratory, Argonne, Illinois, to examine the transformation at the border of the copper matrix and the whitlockite grains (Fig. 1 and Supplementary Fig. 3). The diffraction images and Rietveld refinements revealed that, at the contact between the Cu matrix and the crystal, up to 36% of the whitlockite was transformed into merrillite (Fig. 2, Table 1, Supplementary Table 1 and Supplementary Data 1). Towards the interior of the grain, the amount of merrillite decreases and the innermost kernel of the former crystal is highly strained polycrystalline whitlockite.

### Shock pressure effect

Experiments GG094 and GG095 were performed to investigate the effect of varying shock pressure on whitlockite to merrillite transformations. Both experiments used whitlockite powder (0.45–10 μm size fraction) as the sample. Peak shock pressure for experiment GG094 was ∼20 GPa, whereas shock pressure in experiment GG095 was much lower at∼7.5 GPa (Table 1). Despite higher peak pressures in experiment GG094, approximately the same whitlockite transformation occurred in experiment GG094 and experiment GG095 (12% versus 11%, respectively) (Table 1 and Supplementary Figs 2 and 4). Transformation may therefore be driven by shock generated heating rather than directly by shock compression.

### Molecular dynamics simulations

**Figure 3:**
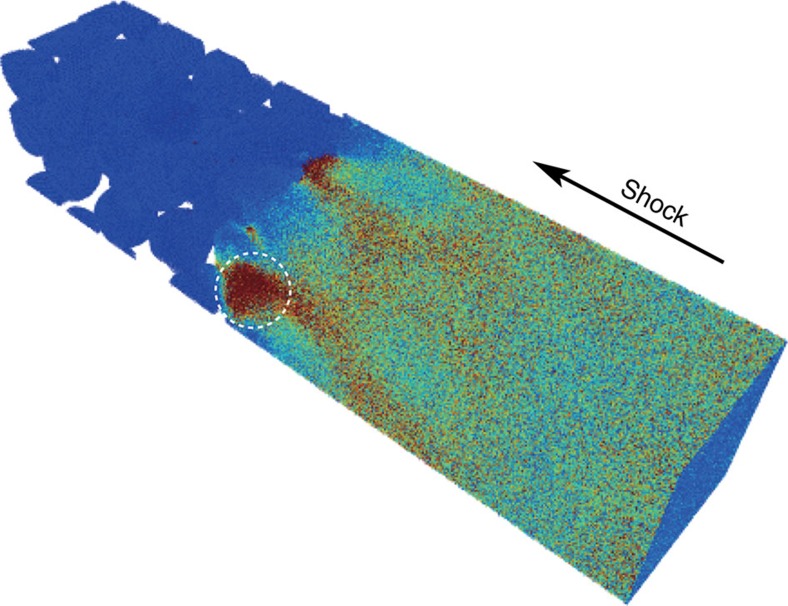
Snapshot of modelled Cu-nanopowder under shock wave loading at 27 GPa. The white dashed circle refers to a hotspot region. Colour coding is based on kinetic energy with red representing highest values.

Molecular dynamics simulations were performed to further investigate conditions relevant to the shock transformation of whitlockite to merrillite. As fundamental data are lacking for phosphate mineral powders, we simulated the effect of different shock strengths (5, 15 and 27 GPa) on temperature using Cu powders similar to the Cu matrix used in experiment GG093. A snapshot of dynamic compression shows hotspot formation at locations associated with void collapse, as indicated by the white circle in Fig. 3 (see also Supplementary Movie 1 for more model output). We performed one- and three-dimensional binning analyses to calculate *T*_bulk_ and *T*_hotspot_, respectively. *T*_bulk_ is the average temperature behind the shock front. *T*_hotspot_ depends on the exact location of a hotspot and its value evolves with time. *T*_hotspot_ temperatures became significantly hotter at high shock strengths (15 and 27 GPa) because of the production of internal jets in front of the shock front, which impacted and heated particles downstream. No jetting was observed in the 5 GPa model run. In all modelled cases, *T*_hotspot_ values exceeded 1,000 °C, the laboratory transformation temperature of whitlockite to merrillite (Table 2). Thus, the simulation is consistent with whitlockite to merrillite transformation upon void collapse.

## Discussion

One of the confounding factors encountered when working with meteorites is the fact that, to some degree, all meteorites have experienced shock that has altered them. Many of the effects of shock on the mineralogy and texture in meteorites are known or obvious in observation (for example, fractures, high-pressure phases, strain textures and amorphized phases). However, there are some effects of shock that are not as easily detectable or quantifiable, including the potential devolatilization of phases^53^,^54^. An understanding of these aspects of shock alteration in meteorites is critical for making accurate interpretations of meteorite or parent body properties. Mineral volatile contents from meteorites, for example, have been used to interpret parent body volatile histories or accretion temperatures^7^,^23^,^55^,^56^. If the volatile content of the interpreted phases is not that of the original parent material and this is not taken into account, misinterpretations can result.

The experimental results of this study conclusively demonstrate the transformation of significant amounts of starting whitlockite to merrillite during laboratory shock experiments. Although the number of experiments is insufficient for establishing a rate law, the data do indicate that less densely packed samples (that is, larger void spaces) exhibit an overall greater extent of transformation (for example, GG092 versus GG094). Transformation also occurs predominantly at grain boundaries between whitlockite and the matrix (Fig. 1, GG093) or near voids (GG092). Whitlockite to merrillite transformation in experiment GG095 is about the same as experiment GG094, despite a shock pressure in GG094 of nearly three times as much. Thus, the results indicate that the transformation from whitlockite to merrillite is likely temperature rather than pressure driven.

Consistent with this observation, our molecular dynamic simulations indicate, as might be expected^57^, temperature enhancement at grain boundaries between materials of different impedance such as whitlockite–copper, generally enhanced temperatures in less densely packed powdered samples and much greater heating during collapse of voids. Molecular dynamic simulations indicate that even rather small voids can generate locally and temporarily high temperatures, such as that circled in Fig. 3 (see also Supplementary Movie 1). All *T*_hotspot_ (local hotspot) values in the modelling well exceed 1,000 °C (Table 2). Such high temperatures are well beyond the temperature necessary for the transformation of whitlockite to merrillite^14^,^15^,^16^, even at low peak pressures, and strongly support whitlockite transformation driven by temperature rather than direct shock compression. The experimentally observed transformation of whitlockite to merrillite in this study opens the possibility that merrillite in some meteorites may have been, in part or in whole, whitlockite before shock and some remnant whitlockite might still exist in meteorites. This would especially hold for some Martian meteorites where late-stage parent magmas are thought to have held ample water at the time of merrillite/whitlockite crystallization^11^,^30^. If this is the case, then some remnant whitlockite might be expected in Martian meteoritic merrillite.

Despite some nomenclature confusion in the literature where the term ‘whitlockite' has been applied to merrillite, true whitlockite has not been verified in meteorites^8^,^14^,^30^. However, because of the similarities between merrillite and whitlockite, few analytical methods can distinguish between the two phases and, as such, during routine meteorite analyses the merrillite endmember is typically just assumed^23^. Previous work by McCubbin *et al*.^30^ using secondary ion mass spectrometry (SIMS) on Shergotty merrillite did detected sufficient H^+^ (196 p.p.m. as H_2_O) to potentially account for a few wt% of whitlockite. SIMS is an ablative technique that analyses into the sample beyond the surface, so it is less susceptible to surface contamination of H^+^ than many other techniques. McCubbin *et al*.^30^ could not confirm the actual phase as whitlockite (or any other), as SIMS is an elemental only technique. However, their H^+^ detections are almost certainly an indicator of a trace/minor H^+^-bearing phosphate phase within Shergotty merrillite.

Formation of additional phosphate-containing phases is also consistent with whitlockite to merrillite transformation. In shock experiment GG092, ∼90% of the whitlockite was transformed to merrillite and an additional Ca-phosphate phase structurally related to hurlbutite. Similarly, Adcock *et al*.^16^ have previously noted the formation of multiple additional trace Ca-phosphate phases within merrillite synthesized by the heating of whitlockite and showed that the trace phases help balance the chemistry deficiency created by transformation of whitlockite to merrillite^16^. The additional trace phase detected in our shock experiment probably plays a similar role and future confirmation of transformation in meteorites could come in the form of further characterization of additional phosphate phases within natural merrillite.

Of course these, or any laboratory-scale shock experiments, cannot reproduce the exact complexity of natural shock events. In particular, the time scale of our experiments is 10^3^–10^5^ times shorter than that of the natural shock states the meteorites have experienced. For example, the shock event that ejected the Martian meteorite Tissint from Mars is estimated to have lasted 10–20 ms (ref. 42). Our experiments generated shock state durations of closer to 10 μs. During an actual natural shock event, the much longer sustained shock state and associated heating would be expected to transform higher amounts of whitlockite to merrillite than in laboratory experiments, potentially reaching complete transformation. In this case, all evidence of the previous whitlockite would be removed and trace Ca-phosphate phases within merrillite, similar to those detected in GG092 or as noted by Adcock *et al*.^16^, might be the only indicator of shock transformation. Even with the presence of such phases, without trace whitlockite, only a returned, unshocked sample of original material could conclusively confirm whitlockite devolatilization.

The possibility of devolatilization of whitlockite to merrillite in meteorites, regardless of extent, has both broad and important implications, in particular for achondritic meteorite interpretations. Volatile contents of minerals in meteorites (for example, F^−^, Cl^−^ and H_2_O) are often used as indicators of parent body volatile budgets and can therefore be used to interpret past process and formation histories of parent bodies (for example, see refs 5, 7, 8, 9, 11, 23, 30, 58) such as mineral and melt thermal stabilities and melt viscosity^35^. The potential transformation of whitlockite to merrillite in meteorites is especially important to interpretations of Mars, where volatile content has implications for magma eruptive processes, including the past degassing of water ultimately available to the planetary surface^9^,^59^,^60^,^61^.

The possible presence of whitlockite rather than merrillite on Mars also has strong astrobiological implications. Phosphorus, as phosphate^62^ or a reduced species^3^, is among the elements considered necessary for life^63^. Because of low solubility, limited reactivity with some organics and the lack of a significant volatile phase, the prebiotic availability of phosphate was likely to be a hurdle for the origin of life on Earth (that is, the ‘phosphate problem')^63^ and potentially on Mars^3^,^63^. Whitlockite has a higher solubility (that is, higher final phosphorus concentrations in solution) than merrillite^1^, which itself has a much higher solubility than fluorapatite, the primary igneous source of phosphate on Earth. Thus, significant whitlockite on Mars would mean more available phosphorus in aqueous environments for any potential prebiotic or biotic reactions.

The implications of original whitlockite in chondritic meteorites are less clear (Supplementary Note 2), but the shock results reported here strongly support the conclusions of McCubbin *et al*.^30^, although for far different reasons (Supplementary Note 1), that merrillite, in any meteorite, should not be used as a sole indicator of parent body water contents. Applying meteoritic volatile contents to parent body volatile interpretations may yield only minima. These results also present a cautionary instance in which shock alteration could affect a meteorite in a very significant way, but leave little or no evidence of the shock effects, leading potentially to petrologic misinterpretations. Our results highlight the strong need for returned samples from planetary parent bodies if we wish to make reliable interpretations of parent body—and in fact—solar system origins.

Although meteorites are, and continue to be, of immense value as samples of our solar system, the results of this study emphasize the shortcomings of using them as substitutes for direct samples, especially of planetary parent bodies. They are not direct substitutes. Although some shock effects in meteorites are obvious and can be considered in interpretations, it is possible that some alteration processes leave no detectable trace. Direct sample return is the only path to obtaining samples of another solar system body not altered by shock.

## Methods

### Mineral syntheses

Whitlockite was synthesized using the methods of Adcock *et al*.^1^,^16^, wherein a solution containing 90 ml of high-purity (18.2 MΩ) water, 1.0 g of laboratory-grade hydroxyapatite (Spectrum, reagent grade) and 0.3 g magnesium nitrate hexahydrate (J.T. Baker, ACS grade) were combined in a 125 ml Parr acid digestion vessel (Parr 4748) with an acid-washed polytetrafluoroethylene liner. Once the solution was mixed, it was acidified to pH <2.8 with concentrated phosphoric acid (Alfa Aesar, ACS grade). The vessel was then sealed and incubated in an oven at 240 °C for 7 days. At the end of 7 days, the vessel was removed from the oven and quenched in a water bath in an effort to prevent any further reaction. After cooling, the vessel was opened and the solution decanted leaving the solids. Solid material was rinsed from the vessel using ethanol, allowed to air dry for 24 h, weighed and inspected by optical microscopy for preliminary phase identification. Impurities were typically hydroxyapatite (Ca_5_[PO_4_]_3_OH) and monetite (CaHPO_4_), and were primarily confined to the <75 μm fraction. Output masses were therefore brush sieved on a 200 mesh screen to remove that size fraction.

Chemistry of the phases was confirmed by electron microprobe wavelength-dispersive spectroscopy carried out in a JEOL JXA-8900 at the UNLV EMiL facility on polished epoxy mounts (Supplementary Table 2). Analysis conditions were 20 keV and 10 nA using a beam of 10 μm diameter. Analyses were standardized using Smithsonian mineral standards of olivine (Mg and Fe) and Durango apatite (Ca and P)^64^, and Harvard (Amelia) albite (Na)^65^.

Merrillite occurs within synthesized whitlockite at a level of 1–5 mass % (refs 14, 16) in different batches. The first shock experiment (GG092) used whitlockite from a synthesis batch characterized in previous work (UNLV Whitlockite batch 2)^16^ but not measured for merrillite content. For this one experiment, we conservatively assumed a merrillite content of 5%. The whitlockite used in the remaining experiments (UNLV Whitlockite batch 4) was examined by synchrotron micro-XRD to confirm phases and phase proportions (Fig. 2) and contained 2.1% merrillite. Synchrotron XRD of the starting materials detected no other impurities (thus, they amount to <0.1 mass %).

For shock experiments GG092, GG094 and GG095, synthetic whitlockite was ground in an agate mortar and pestle, and the <10 μm fraction was obtained through suspension in ethanol in a large graduated cylinder and the application of Stokes's settling law. The solution with suspension was decanted and run through a 0.45 μm filter and dried to obtain the 0.45–10 μm fraction. Shock experiment GG093 used crystals embedded in a copper powder matrix to better approximate the matrix in an actual rock (discussed further below).

### Shock experiments

Shock experiments were performed using a single-stage light–gas gun at the Department of Geoscience, Tschauner Group Shock Laboratory, UNLV. Compressed He gas was used to launch the projectiles. The projectiles were flat polished metal flyer plates mounted on Lexan sabots. In each shock experiment, the flyer plate imparted a shockwave into a driver plate of the same material and, through impedance match between driver plate and sample, a shock wave was transmitted into the sample. Either stainless steel (experiment GG095) or rhenium (all other experiments) was used as flyer and driver materials depending on the desired experimental peak pressures (Supplementary Table 1). Before the experiment, the sample was encapsulated in a recovery chamber formed by a front driver plate, a rear plate of the same metal and an annular ring of the same metal between front and rear driver, to confine the sample. This assembly was rigidly mounted in a cylindrical holder of stainless steel. The assembly permitted reverberative shock between the front- and rear-driver plates that sandwich the sample. In one experiment (GG093), five whitlockite single crystal grains of 50 μm diameter were embedded in powdered copper of 1–2 μm grain size with ∼50% calculated porosity. Copper with 50% porosity has an average shock impedance comparable to shergottitic rocks^49^,^50^,^51^,^52^. In a second series of experiments, powdered synthetic whitlockite was loaded into a recovery chamber with porosities of 24–25% calculated by comparing sample density to whitlockite density. Flyer plate velocities were measured with an optical velocimeter. Shock pressures, duration, and velocities were calculated from flyer plate velocities, the impedances of flyers, drivers and their thicknesses using the impedance match equations^36^ (Supplementary Table 1). The shock impedance is determined by the elastic properties and density of the minerals once they are exposed to a shockwave^66^.

### Synchrotron micro-XRD

After the shock experiments, samples were retrieved for examination by synchrotron XRD. Recovered samples GG092, GG094 and GG095 were powders from which aliquots were taken for analysis. Sample GG093 was recovered as a fused piece and polished before analysis.

Sample GG092 was analysed at beamline 16-BM-D (GeoScienceEnviro Center for Advanced Radiation Sources) at the APS by synchrotron powder-XRD. The sample was mounted on a quartz-glass capillary of 30 μm diameter and centered on the ϕ rotation axis. Beam wavelength was 0.485946 Å. Beam was focused using Kirkpatrick–Baesz mirrors vertically and horizontally, and a MAR345 image detector plate was used. Sample GG093 was analysed at both the 16-BM-D (GeoScienceEnviro Center for Advanced Radiation Sources) and the 13-ID-E High Pressure Collaborative Access Team beamlines at APS by synchrotron micro-XRD and synchrotron X-ray fluorescence mapping (13-ID-E only). Beam wavelengths were 0.485946 Å (16-BM-D) and 0.619900 Å (13-ID-E). Diffraction data were collected with a Rayonix X-ray charge-coupled device detector (13-ID-E) or a MAR345 image plate (16-BM-D). Beam was focused using Kirkpatrick–Baesz mirrors vertically and horizontally. Samples GG094 and GG095 were analysed at the 12.2.2 bending magnet beamline of the Advanced Light Source, Lawrence Berkeley National Laboratory, University of California, Berkeley. Both were analysed by synchrotron powder XRD. Samples were mounted on quartz glass capillaries of 30 μm diameter and centred on the *ϕ* rotation axis. Beam wavelength of 0.496070 Å. Beam was focused using Kirkpatrick–Baesz mirrors vertically and horizontally, and a MAR345 image detector plate was used. Sample-detector distances and geometric distortion of diffraction images for all diffraction data were quantified and corrected using GSE-ADA^67^. Diffraction images were integrated using Dioptas^68^. Diffraction signal of merrillite and merrillite-whitlockite mixtures were quantitatively evaluated using the Rietveld method for phase quantification, unit cell and structure refinement. Matches between observed and calculated diffraction patterns based on the fully weighted structure models of merrillite and whitlockite guaranteed unique identification of these two phases. The refinements were evaluated by the weighted refinement factor *R*_wp_ and all converged excellently (Supplementary Table 1). Additional method details are as described in Tschauner *et al*.^69^

### Modelling of bulk and hotspot temperatures during shock

To investigate bulk temperature (*T*_bulk_) and hotspot temperature (*T*_hotspot_) in Cu powders under shock loading, we performed molecular dynamics simulations of Cu nanopowders at different shock strengths. Nanoscale grain size was chosen because of the limitations in number of atoms for simulating a Cu powder aggregate with voids. The initial porosity of the nanopowder was about 50%, consisting of randomly oriented Cu particles with a diameter of 11 nm. The system size was about six million atoms. An accurate embedded atom method potential was used to describe the atomic interactions in Cu^70^. The initial configuration was equilibrated at 300 K and zero pressure, and then subjected to shock wave loading using a moving rigid piston. Different piston velocities were explored, corresponding to different shock pressures, including 5, 15 and 27 GPa (Supplementary Table 1).

It is possible to calculate *T*_bulk_ analytically, or to simulate *T*_bulk_ and *T*_hotspot_ using a continuum code. However, such calculations and simulations vary greatly depending on model parameters and assumptions made, leading to very high estimate uncertainties. Molecular dynamics simulations, given an accurate Cu potential, produce reasonable estimate of these temperatures. Unfortunately, there are no comparably reliable potentials of phosphates for such simulations.

### Data availability

The authors declare that the data supporting the findings of this study are available within the article, its citations, Supplementary Information files and/or from the corresponding author upon request.

## Additional information

**How to cite this article:** Adcock, C. T. *et al*. Shock-transformation of whitlockite to merrillite and the implications for meteoritic phosphate. *Nat. Commun.* **8,** 14667 doi: 10.1038/ncomms14667 (2017).

**Publisher's note:** Springer Nature remains neutral with regard to jurisdictional claims in published maps and institutional affiliations.

## Supplementary Material

Supplementary Information Supplementary Figures, Supplementary Tables, Supplementary Notes and Supplementary References

Supplementary Movie 1 Molecular Dynamics Simulation of Cu-nanopowder under shock wave loading at 27 GPa

Supplementary Data 1 PowderCell 2.2 receipts of the x-ray data refinements used in the study

## Figures and Tables

**Table 1:** Experimental conditions.

**Experiment ID**	**GG092**	**GG093**	**GG094**	**GG095**
Sample starting composition	<10 μm Batch 2 Whit.	Batch 4 Whit.+ copper	<10 μm Batch 4 Whit.	<10 μm Batch 4 Whit.
Whitlockite (%)	95	98	98	98
Merrillite (%)	5*	2	2	2
Peak shock pressure (GPa)	19±2	19±2	20.2±0.5	7.5±0.5
*Fitted post shock composition*				
Whitlockite (%)	11	64	88	89
Merrillite (%)	42	36	12	11
Unconfirmed† (%)	48			

Whit., Mg-whitlockite, synthetic. Batch 2 and Batch 4 refer to large batches of synthetic Mg-whitlockite.

^*^Conservative upper estimate based on measured merrillite content of synthetic whitlockite^14^,^16^.

^†^Previously unknown phase with a structure related to hurlbutite.

**Table 2:** Bulk temperature and hotspot temperature for different shock pressures simulated by molecular dynamics modelling.

***P*** **(GPa)**	***T***_**bulk**_ **(K)**	***T***_**hotspot**_ **(K)**
5	1,000±100	1,350±100
15	1,800±200	4,200±500
27	3,000±200	5,700±500
