# Supplementary material for: Shock-transformation of whitlockite to merrillite and the implications for meteoritic phosphate
Source: Nat Commun. 2017 Mar 6;8:14667. doi: 10.1038/ncomms14667 (PMC5343502; doi:10.1038/ncomms14667)
Supplement: Supplementary Information — Supplementary Figures, Supplementary Tables, Supplementary Notes and Supplementary References [file ncomms14667-s1.pdf]

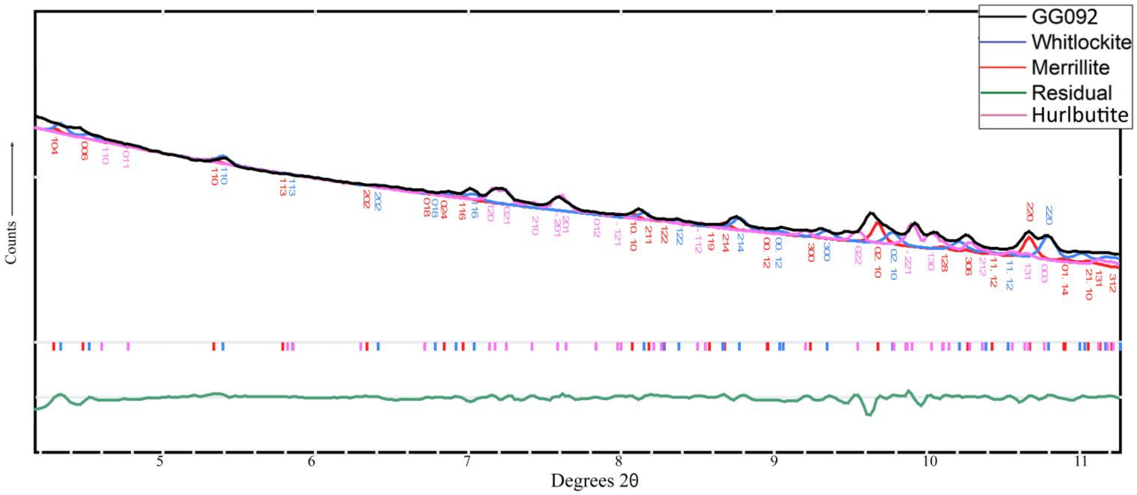

Supplementary Figure 1. Synchrotron micro-XRD of GG092 sample. Rietveld fitting of 10.6% whitlockite (blue pattern), 41.6% merrillite (red pattern), and 47.8% hurlbutite-related phase (magenta).

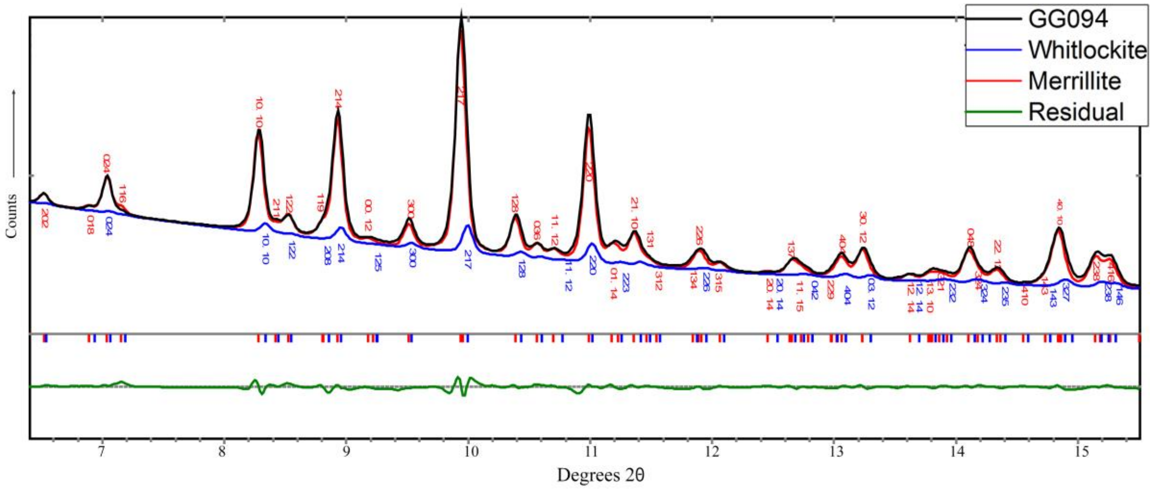

Supplementary Figure 2. **Synchrotron micro-XRD of sample GG094**. Rietveld fitting of 87.9% whitlockite (blue pattern), and 12.1% merrillite (red pattern)

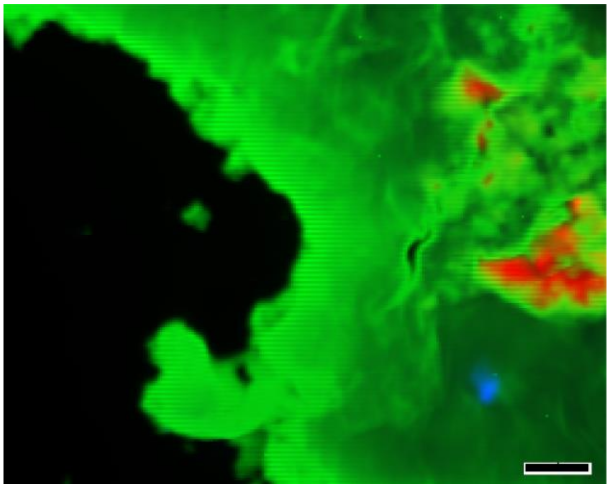

Supplementary Figure 3. **XRF map of recovered sample GG0093 showing a whitlockite single crystal grain in copper matrix (raw data map).** Red = Ca (indicating whitlockite/merrillite) Green = copper. At the contact between matrix and the crystal ~35% of whitlockite has been transformed into merrillite based on Rietveld refinements. Further inward the amount of merrillite is smaller and the innermost kernel of the former crystal is highly strained polycrystalline whitlockite. Subsequent diffraction images were taken in a grid scan over the exposed phosphate grains. 1 pixel = 2×2 µm². The XRF map was taken at beamline13-IDE at the APS. A blue/red color adjusted (Technicolor 2-strip LUT replacement) version of this image to avoid red/green combinations appears in the accompanying manuscript. Scale bar is 50 µm.

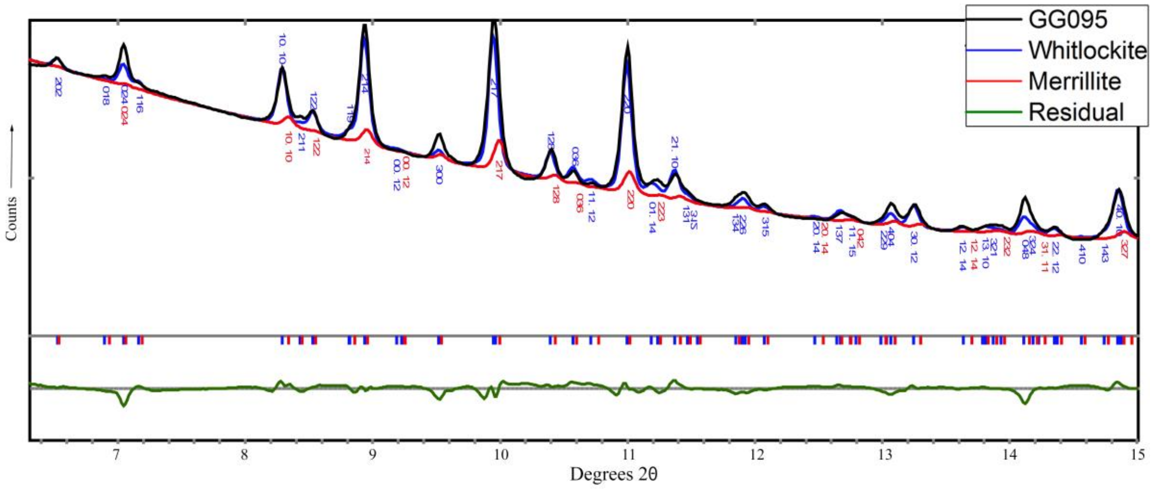

Supplementary Figure 4. Synchrotron micro-XRD of GG095 sample. Rietveld fitting of 88.9% whitlockite (blue pattern), and 11.2% merrillite (red pattern).

SUPPLEMENTARY TABLES

Supplementary Table 1. Experimental Conditions

Experiment ID	GG092	GG093	GG094	GG095
**Sample Starting Composition**	<10 µm Batch2 Whit.	Batch 4 Whit.+ Cu powder[†]	<10 µm Batch 4 Whit.	<10 µm Batch 4 Whit.
**Whitlockite (%)**	95	98	98	98
**Merrillite (%)**	5[§]	2	2	2
**Porosity (%)**	25	50*	24	24
**Flyer/Driver Material**	Rhenium	Rhenium	Rhenium	Stainless Steel
**Flyer Velocity (m/s)**	800	700	700	770
**Peak Shock Pressure (GPa)**	19 ± 2	19 ± 2	20.2 ± 0.5	7.5 ± 0.5
**Fitted post shock composition**				
**Whitlockite (%)**	10.6	64.4	87.9	88.9
**Merrillite (%)**	41.6	35.6	12.1	11.2
**Unidentified(%)**	47.8[¥]			
**Rietveld refinement goodness of fit: R_{wp}**	1.97	9.30	1.75	1.86

Whit. = Mg-whitlockite, synthetic. Cu = copper powder. Batch 2 and Batch 4 refer to large batches of synthetic Mg-whitlockite.
[§]Conservative estimate based on typical content of synthesized whitlockite[1,2].
[¥]Previously unknown phase with a structure related to (and fit with) hurlbutite.
[†]Shock impedances for whitlockite (lower than the Cu-matrix) were bracketed using the compressibility and pressure derivative of synthetic tuite[3] and apatite[4].
*Based on copper.

Supplementary Table 2. Average electron microprobe analysis of synthetic whitlockite Mg-whitlockite used in shock experiments.

	Mg-Whitlockite[a]		Mg-Whitlockite[b]	
**Oxide**	**Wt. %**	**S.D.**	**Wt. %**	**S.D.**
**Na_2O**	0.01	0.06	B.D.	N/A
**FeO**	0.01	0.03	0.02	0.03
**CaO**	47.69	0.20	47.61	0.20
**MgO**	3.72	0.09	3.54	0.14
**MnO**	B.D.	N/A	B.D.	N/A
**P_2O_5**	47.15	0.61	46.85	0.27
**Cl-**	0.01	0.01	0.01	0.01
**SO_3**	0.16	0.05	0.16	0.05
**H_2O***	0.86	N/A	0.86	N/A
**Total**	99.61		98.02	

N/A = not applicable. S.D. = Standard Deviation. B.D. = Below Detection Limit
*Water based on ideal whitlockite[1]
[a]Whitlockite used in experiments GG093, GG094, and GG095 (UNLV Batch 4). Calculated stoichiometry = $Ca_{8.97}(Mg_{0.98})(PO_3OH)_{1.01}(PO_4)_{6.00}$. Average based on 11 analyses.
[b]Whitlockite used in experiment GG092 (UNLV Batch 2). Calculated stoichiometry = $Ca_{9.0}(Mg_{0.9})(PO_3OH)_1(PO_4)_6$ from Adcock et al, 2014[2]. Average based on 17 analyses.

Supplementary Note 1

McCubbin et al., (2014)[5] considered the question of why whitlockite didn't form in martian meteorites as it seems that whitlockite should form at least in solid solution in the parent magmas they had investigated:

> *"Based on the OH abundances in apatite, the residual Shergotty melt would have had 1.5–3.0 wt% H2O at the time of phosphate crystallization (McCubbin et al. 2012)[5]. If merrillite and whitlockite form a solid solution (as demonstrated by Hughes et al. 2008)[1], it seems reasonable that the merrillite forming from this H-rich liquid should have a larger whitlockite component than 0.5–2.5%."*

This was based in part in part on OH^- partition coefficients that strongly suggested significant OH-bearing chlorapatite would be an indicator of abundant H_2O in magmas at the time of phosphate mineral formation[6,7]:

> *"Based on recent apatite-melt partitioning experiments on shergottitic liquids, the halogens play a much larger role in stabilizing magmatic apatite than H. In fact, apatite prefers F over OH by a factor of approximately 100, and it prefers Cl over OH by a factor of about 20 (Boyce et al. 2014; McCubbin et al. 2013c, 2014; Vander Kaaden et al. 2012)[8-11]."*

They ultimately suggested that limited thermal stability of H^+ in whitlockite might promote the formation of merrillite over whitlockite in magmas. Since whitlockite can be devolatilized at <1050 °C, a temperature close to the final crystallization temperatures of martian magmas, then whitlockite may not be able to form. Thus, merrillite should not be used as an indicator of water content.

However, this devolatilization temperature is based on laboratory experiments done open to dry air at 1 atm, a setting much different from a late stage martian magma. Adcock et al., (2013)[2] noted incomplete whitlockite to merrillite transformations at 1100°C in sealed SiO_2 tubes, suggesting water fugacity (which was increasing during transformation in those experiments) plays a determinant role in phase stability. Thus, in a setting where chlorapatite OH^- contents suggest high late-stage magma water fugacity[e.g. 5], it is not conclusively clear why the anhydrous end-member merrillite occurs rather than whitlockite or a mix in solid-solution.

Supplementary Note 2

The specific implications to chondrites of potential whitlockite to merrillite transformation are not as clear as for other meteorites. Chondritic meteorite parent bodies formed during the very early stages of our solar system and never differentiated. For this reason, they are very important to studies of solar system origin, especially the distribution of water in the early solar system[12]. However, despite their undifferentiated state, they are not homogeneous and can vary dramatically. Enstatite chondrites, for instance, represent some of the driest rocks in the solar system and are more reduced than the other chondrites. As a result, phosphate minerals are rare in these meteorites and instead, more reduced phosphide minerals occur such as schreibersite[13]. Ordinary chondrites possess both merrillite and apatite, however, these minerals are the results of thermal metamorphism rather than igneous processes[14,15]. In addition, unlike

the OH-bearing chlorapatite found in shergottites that raised questions about merrillite interpretations in martian meteorites[5], the chlorapatite in the meteorites is extremely dry, suggesting that conditions during the metamorphism event that created the apatite and merrillite were dry in nature and not conducive to whitlockite formation in the first place[14]. In contrast to many other chondrites, carbonaceous chondrites are not dry, but can contain 5-10% water by mass[12]. These meteorites likely had abundant water during phosphate formation, are among the least shocked, and have a D/H ratio similar to that of Earth, suggesting very little perturbation of the water in these meteorites. These chondrites may be one of the best candidates for future study directed at detecting potential whitlockite.

Supplementary References.

1 Hughes, J. M., Jolliff, B. L. & Rakovan, J. The crystal chemistry of whitlockite and merrillite and the dehydrogenation of whitlockite to merrillite. *American Mineralogist* **93**, 1300-1305, doi:10.2138/am.2008.2683 (2008).

2 Adcock, C. T., Hausrath, E. M., Forster, P. M., Tschauner, O. & Sefein, K. J. Synthesis and characterization of the Mars-relevant phosphate minerals Fe- and Mg-whitlockite and merrillite and a possile mechanism that maintains charge balance during whitlockite to merrillite transformation. *American Mineralogist* **99**, 1221-1232 (2014).

3 Zhai, S., Liu, X., Shieh, S. R., Zhang, L. & Ito, E. Equation of state of γ-tricalcium phosphate, γ-Ca3 (PO4) 2, to lower mantle pressures. *American Mineralogist* **94**, 1388-1391 (2009).

4 Brunet, F. *et al.* Compressibility and thermal expansivity of synthetic apatites, Ca 5 (PO 4) 3 X with X= OH, F and Cl. *European Journal of Mineralogy* **11**, 1023-1035 (1999).

5 McCubbin, F. M. *et al.* Volatile abundances of coexisting merrillite and apatite in the martian meteorite Shergotty: Implications for merrillite in hydrous magmas. *American Mineralogist* **99**, 1347-1354 (2014).

6 Mathez, E. A. & Webster, J. D. Partitioning behavior of chlorine and fluorine in the system apatite-silicate melt-fluid. *Geochimica et Cosmochimica Acta* **69**, 1275-1286 (2005).

7 McCubbin, F. M. *et al.* Experimental investigation of F, Cl, and OH partitioning between apatite and Fe-rich basaltic melt at 1.0–1.2 GPa and 950–1000 C. *American Mineralogist* **100**, 1790-1802 (2015).

8 McCubbin, F. *et al.* in *Lunar and Planetary Science Conference.* 2741.

9 Vander Kaaden, K., McCubbin, F., Whitson, E., Hauri, E. & Wang, J. in *Lunar and Planetary Science Conference.* 1247.

10 Boyce, J., Tomlinson, S., McCubbin, F., Greenwood, J. & Treiman, A. The lunar apatite paradox. *Science* **344**, 400-402 (2014).

11 McCubbin, F., Vander Kaaden, K., Whitson, E., Bell, A. & Shearer, C. in *Lunar and Planetary Science Conference.* 2748.

12 Alibert, Y. *et al.* Origin and formation of planetary systems. *Astrobiology* **10**, 19-32 (2010).

13 Fuchs, L. H. in *Meteorite research* 683-695 (Springer, 1969).

14 Jones, R. H. *et al.* Phosphate minerals in LL chondrites: A record of the action of fluids during metamorphism on ordinary chondrite parent bodies. *Geochimica et Cosmochimica Acta* **132**, 120-140 (2014).

15 Brearley, A. J. & Jones, R. H. Chondritic meteorites. *Reviews in Mineralogy and Geochemistry* **36**, 3.1-3.398 (1998).
